# p53 attenuates AKT signaling by modulating membrane phospholipid composition

**DOI:** 10.18632/oncotarget.4067

**Published:** 2015-06-03

**Authors:** Natalia Rueda-Rincon, Katarzyna Bloch, Rita Derua, Rajesh Vyas, Amy Harms, Thomas Hankemeier, Niamat Ali Khan, Jonas Dehairs, Muralidhararao Bagadi, Maria Mercedes Binda, Etienne Waelkens, Jean-Christophe Marine, Johannes V. Swinnen

**Affiliations:** ^1^ KU Leuven - University of Leuven, Department of Oncology, Laboratory of Lipid Metabolism and Cancer, Leuven, Belgium; ^2^ KU Leuven - University of Leuven, Department of Cellular and Molecular Medicine, Laboratory of Protein Phosphorylation and Proteomics, Leuven, Belgium; ^3^ KU Leuven - University of Leuven, Center for the Biology of Disease, Laboratory for Molecular Cancer Biology, VIB, Leuven, Belgium; ^4^ KU Leuven - University of Leuven, Department of Human Genetics, Laboratory for Molecular Cancer Biology, VIB, Leuven, Belgium; ^5^ Division of Analytical Biosciences, Leiden Academic Centre for Drug Research, Leiden University, The Netherlands; ^6^ Netherlands Metabolomics Centre, Leiden, The Netherlands; ^7^ Institut de Recherche Expérimentale et Clinique (IREC), Pôle de Gynécologie, Bruxelles, Belgium

**Keywords:** p53, SCD, SREBP, phospholipids, cancer

## Abstract

The p53 tumor suppressor is the central component of a complex network of signaling pathways that protect organisms against the propagation of cells carrying oncogenic mutations. Here we report a previously unrecognized role of p53 in membrane phospholipids composition. By repressing the expression of stearoyl-CoA desaturase 1, SCD, the enzyme that converts saturated to mono-unsaturated fatty acids, p53 causes a shift in the content of phospholipids with mono-unsaturated acyl chains towards more saturated phospholipid species, particularly of the phosphatidylinositol headgroup class. This shift affects levels of phosphatidylinositol phosphates, attenuates the oncogenic AKT pathway, and contributes to the p53-mediated control of cell survival. These findings expand the p53 network to phospholipid metabolism and uncover a new molecular pathway connecting p53 to AKT signaling.

## INTRODUCTION

Since its discovery in 1979 [1, 2], p53 has emerged as one of the most important tumor suppressors. It is lost or mutated in more than 50% of all human cancers and there is increasing evidence that, although wild-type, p53 function is compromised in the remaining cancers [3]. In response to a variety of cellular stress signals such as DNA damage or oncogene activation, p53 functions as a transcription activator inducing a complex network of hundreds of genes which in turn trigger context-dependent antiproliferative cellular responses including cell cycle arrest, apoptosis, autophagy and senescence [3–5]. This network encompasses direct effectors of these processes, including the cyclin-dependent kinase inhibitor 1A (p21), the p53 upregulated modulator of apoptosis (PUMA) and the BCL2-associated X protein (BAX), but also involves many indirect effects and cross-talks with (proto-)oncogenes and other tumor suppressors [6]. Through these cross-talks, the p53 network forms ramifications to numerous other cellular processes including cell migration, angiogenesis and cell metabolism [7–10]. All of these p53-dependent activities coordinately adapt cellular functioning in pro-oncogenic stress conditions and protect normal cells from becoming malignant.

Cell membranes, which are primarily composed of phospholipids, play a central role in many cellular processes, several of which are also affected by p53. Phospholipids not only have a major structural function in the generation of biological membranes but are also implicated in the synthesis of a series of messenger molecules involved in crucial signaling pathways. There is evidence that changes in the fatty acyl composition of membrane phospholipids potently affect membrane properties such as membrane fluidity and dynamics, and result in changes in membrane functionality [11–13]. Not surprisingly, several alterations in membrane lipid metabolism and lipogenic enzymes have been observed in cancer cells [14–16]. These changes affect cell proliferation and survival, they attenuate the sensitivity of cancer cells to chemotherapeutics and oxidative damage, they protect cancer cells from endoplasmic-reticulum (ER) stress and lipotoxicity, and they play a role in migration, invasion and tumor angiogenesis [17–21]. Hence, interference with lipid metabolism may have potential for antineoplastic intervention [22–24].

In view of this central role of membrane phospholipids in cellular functioning, the aim of this study was to explore the impact of p53 on the phospholipid composition of cellular membranes and its role on cancer-related pathways. We demonstrate that p53 elicits profound changes in phospholipids, particularly in their degree of mono-unsaturation. These changes result in the attenuation of the oncogenic AKT pathway and through this route, in the effects of p53 on cell survival. These findings uncover a key role for phospholipid metabolism as a mediator in the crosstalk linking two of the most important cancer-relevant pathways.

## RESULTS

### Activation of p53 expression evokes changes in phospholipid profiles both *in vitro* and *in vivo*

**Figure 1:**
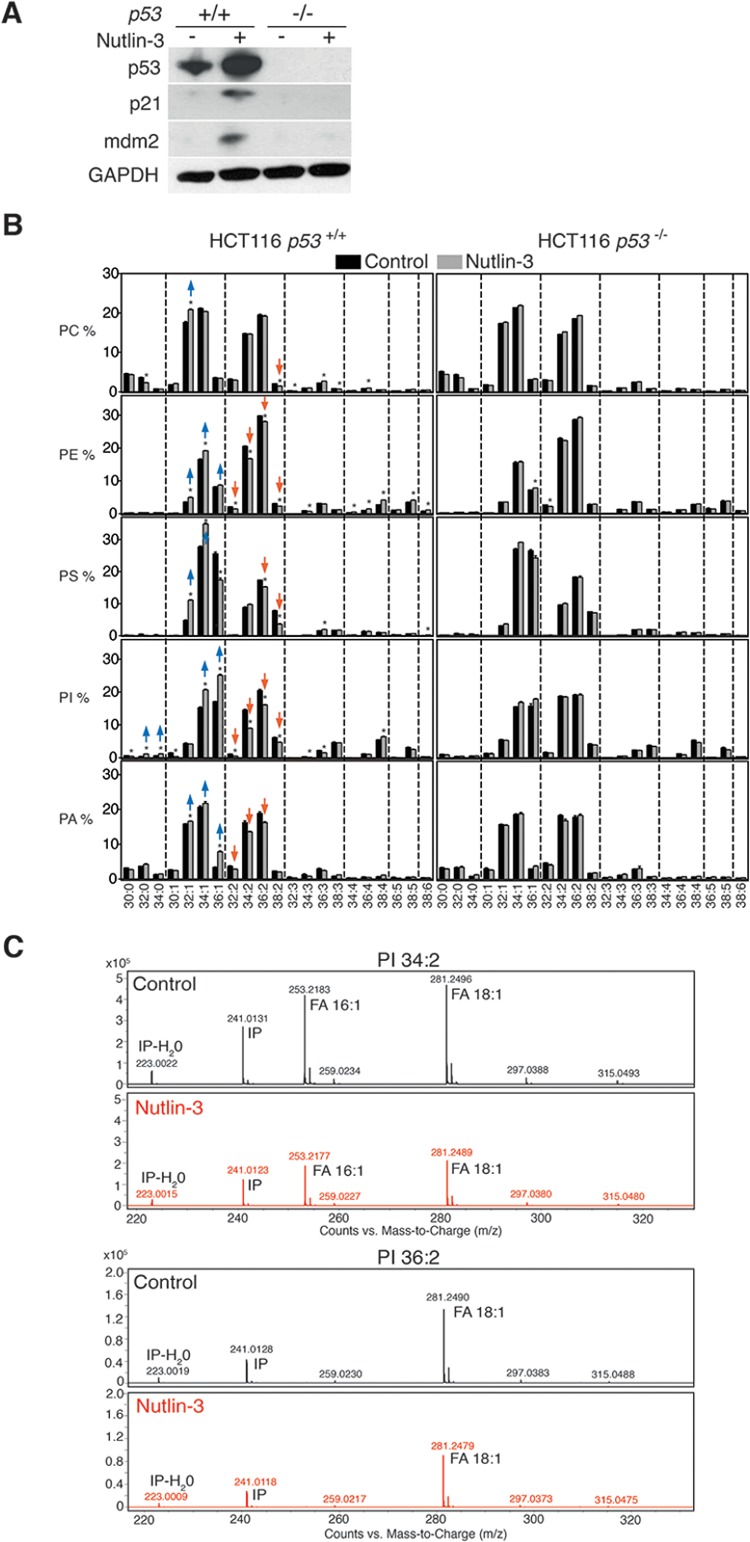
p53 activation reduces mono-unsaturation of phospholipids in cancer cells **A.** Activation of p53 by nutlin-3. HCT116 *p53*^+/+^ and *p53*^−/−^ cells were treated with 5 μM nutlin-3 for 72 h. Expression levels of p53, p21 and MDM2 were determined by western blotting. **B.** Effect of nutlin-3 on the abundance of intact phospholipid species of HCT116 *p53*^+/+^ and *p53*^−/−^ cells (Phosphatidylcholine (PC), Phosphatidylethanolamine (PE), Phosphatidylserine (PS), Phosphatidylinositol (PI) and Phosphatidic acid (PA)). The graph shows the relative abundance of the different measured lipid species in nutlin-3-treated and control cells. Lipid species are ranked based on the number of unsaturations in both fatty acyl chains combined, and within each unsaturation subgroup, based on the total carbon number. *p < 0.05 by multiple *t* test and Holm-Sidak correction for multiple comparisons. Data are presented as means ± SEM **C.** Acyl chain composition of PI 34:2 and PI 36:2 lipid species extracted from control and nutlin-3 treated cells. IP: inositol phosphate ion.

To establish a link between p53 biology and phospholipid composition, we exposed *p53*-wild-type HCT116 cells to nutlin-3 [25], a potent and specific antagonist of the main p53 negative regulator, MDM2 (murine double minute 2). Expression levels of p53 and its targets p21 and MDM2 were increased upon nutlin-3 treatment (Figure 1A). Isogenic *p53* knockout (*p53*^−/−^) cells were used as controls. Seventy-two hours after nutlin-3 exposure, lipids were extracted and subjected to targeted shotgun lipidomics [19]. Several significant changes were observed in the relative abundance of phospholipid species (Figure 1B). Among the most prominent changes was a decrease in phospholipid species with two unsaturations (in both acyl chains together) such as 32:2, 34:2, 36:2 and 38:2, concomitantly with an increase in lipid species with one or no acyl chain unsaturations such as 32:1, 34:1, 36:1, 32:0 and 34:0. These changes were most outspoken in the phosphatidylinositol (PI) headgroup class. Similar changes in lipid profiles were observed when p53 was activated in an alternative way in response to genotoxic stress induced by doxorubicin instead of nutlin-3 treatment (Supplementary Figure S1).

MS/MS analysis of PI species with two unsaturations like PI 34:2 and PI 36:2 revealed that these phospholipids were mainly composed of two mono-unsaturated acyl chains (C16:1 and C18:1) and confirmed the decrease in these species upon nutlin-3 treatment (Figure 1C). These findings indicate that p53 induced a shift of phospholipids with two mono-unsaturated acyl chains towards those with one or no mono-unsaturated acyl chains. p53-deficient cells did not exhibit these changes upon nutlin-3 treatment, confirming the strict p53-dependency of these effects.

**Figure 2:**
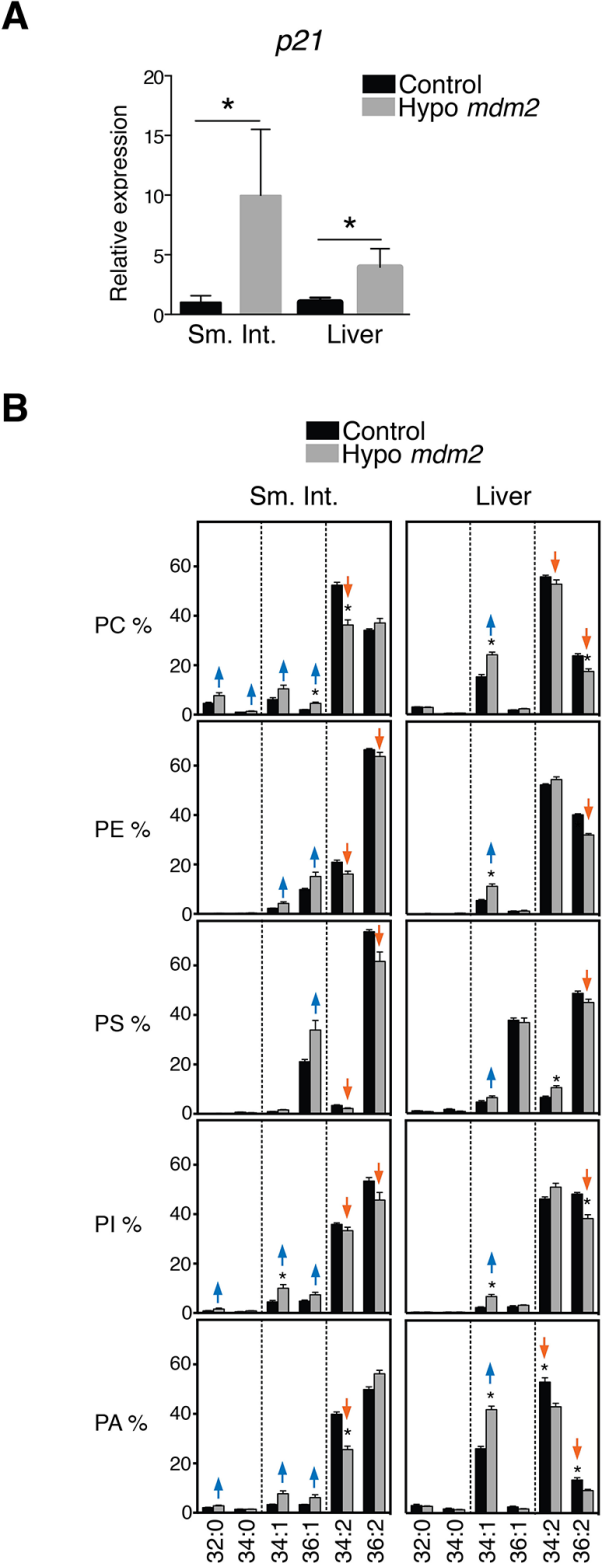
p53 expression causes changes in phospholipid profiles in tissues of *Mdm2* mice **A.** p53 activation in hypo *Mdm2* mice. *p21* mRNA expression in small intestine (Sm. Int.) and liver in hypo *Mdm2* and control (*n* = 3–5). Levels are shown in fold relative to control mice. *p < 0.05, Mann-Whitney test. **B.** Analysis of PC, PE, PS, PI and PA species in the small intestine and liver from hypo *Mdm2* and control mice (*n* = 4–5). Graphs are organized as explained in the legend to Figure 1. *p < 0.05 by multiple *t* test and Holm-Sidak correction for multiple comparisons.

To study the ability of p53 to influence lipid composition *in vivo*, we collected tissues from mice expressing hypomorphic levels of MDM2 [26] (Figure 2A). Although viable, these mice exhibit a series of p53-dependent phenotypes such as lymphopenia, radiosensitivity and increased levels of spontaneous apoptosis in the thymus, spleen and intestine. Changes in lipid composition observed in several tissues of these mice are similar to those in HCT116 cells; a significant decrease in phospholipids with two unsaturations such as 34:2 and 36:2 and an increase in species with only one unsaturation was particularly evident in small intestine and to a lesser extent in liver (Figure 2B).

### p53-induced changes in mono-unsaturation of phospholipid acyl chains are mediated by repression of SCD (stearoyl-CoA desaturase 1) expression

**Figure 3:**
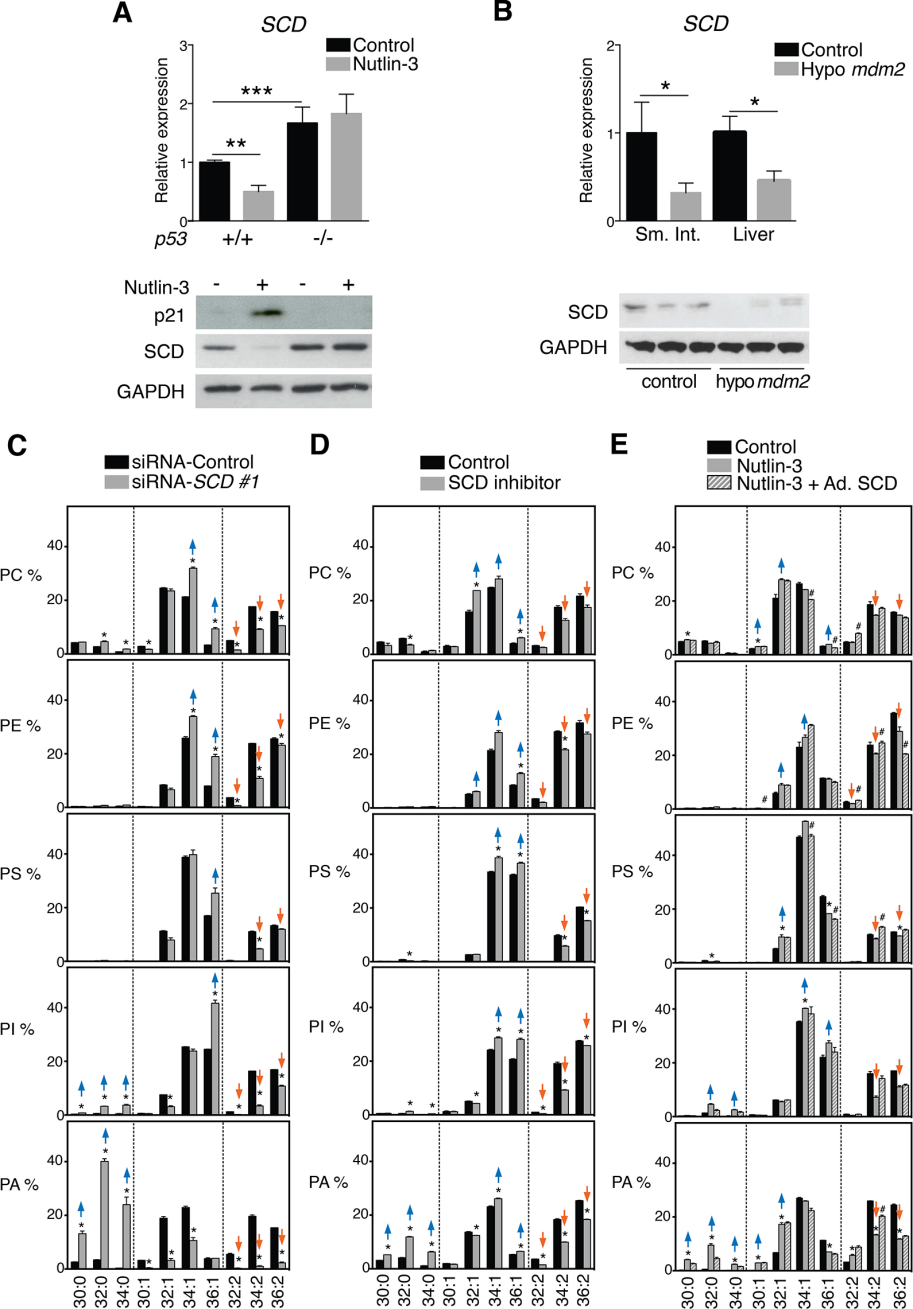
p53-induced changes in phospholipid profiles are mediated by repression of SCD expression **A.** p53 activation affects SCD expression. HCT116 *p53*^+/+^ and *p53*^−/−^ cells were treated with 5 μM nutlin-3 for 72 h. *SCD* mRNA levels were assessed by RT-qPCR and expressed relative to *p53*^+/+^ cells in the control condition; **p < 0.01, ***p < 0.001 by ANOVA. SCD protein levels were assessed by western blotting. P21 and GAPDH were used as positive and loading control, respectively. **B.** Effect of p53 activation on SCD *in vivo*. *Scd* mRNA levels were analyzed by RT-qPCR in small intestine (Sm. Int.) and liver of hypo *Mdm2* and control mice (*n* = 3–5). Levels are expressed relative to control mice. *p < 0.05, Mann-Whitney test. Representative western blot of liver of 3 control and 3 hypo *Mdm2* mice. **C** and **D.** Effect of KD or inhibition of *SCD* on phospholipid profiles. HCT116 cells were transfected with siRNA *SCD* or treated with 40 nM SCD inhibitor. After 72 h, cell pellets were collected for lipid analysis. **E.** Reintroduction of *SCD* in nutlin-3-treated cells restores phospholipid profiles. HCT116 *p53*^+/+^ cells were infected with an adenovirus encoding *SCD* or control virus. 4 h after infection, cells were treated with nutlin-3 for 72 h and collected for phospholipid analysis. Graphs are organized as explained in the legend to Figure 1. *p < 0.05 (control vs nutlin-3) and #*p* < 0.05 (nutlin-3 vs nutlin-3 + Ad. SCD) by multiple *t* test and Holm-Sidak correction for multiple comparisons.

To gain insight into the mechanisms underlying p53-mediated mono-unsaturation of phospholipid acyl chains, we examined the effect of p53 activation on SCD expression, the main enzyme involved in the synthesis of mono-unsaturated fatty acids [27]. RT-qPCR and western blot analyses revealed that this enzyme was significantly down-regulated in nutlin-3-exposed *p53*^+/+^ HCT116 cells. Nutlin-3 did not decrease SCD expression in *p53*^−/−^ cells, which showed elevated SCD steady state levels consistent with the ability of p53 to repress SCD expression (Figure 3A). SCD levels were also significantly decreased in tissues from *Mdm2* hypomorphic mice (Figure 3B). To confirm the involvement of SCD in acyl chain mono-unsaturation observed upon p53 activation, we knocked-down *SCD* in HCT116 cells with two independent siRNAs (Supplementary Figure S2). Similar to nutlin-3 treatment, SCD knock-down induced a shift of phospholipids with two mono-unsaturated acyl chains towards those with one or no mono-unsaturated acyl chains (Figure 3C and Supplementary Figure S3). The shift was even more pronounced than this induced by nutlin-3. Hence, the extent of *SCD* KD was greater in these experimental conditions. Similar effects were observed after chemical inhibition of SCD (Figure 3D). Furthermore, restoration of SCD expression in nutlin-3-treated cells using an adenoviral-based approach ablated the p53-dependent effect on the phospholipids composition (Figure 3E). Together these data establish SCD as a key mediator of the effects of p53 on lipid metabolism.

### Decreased SCD expression by p53 is mediated by repression of SREBP1c (sterol regulatory element binding protein-1c)

**Figure 4:**
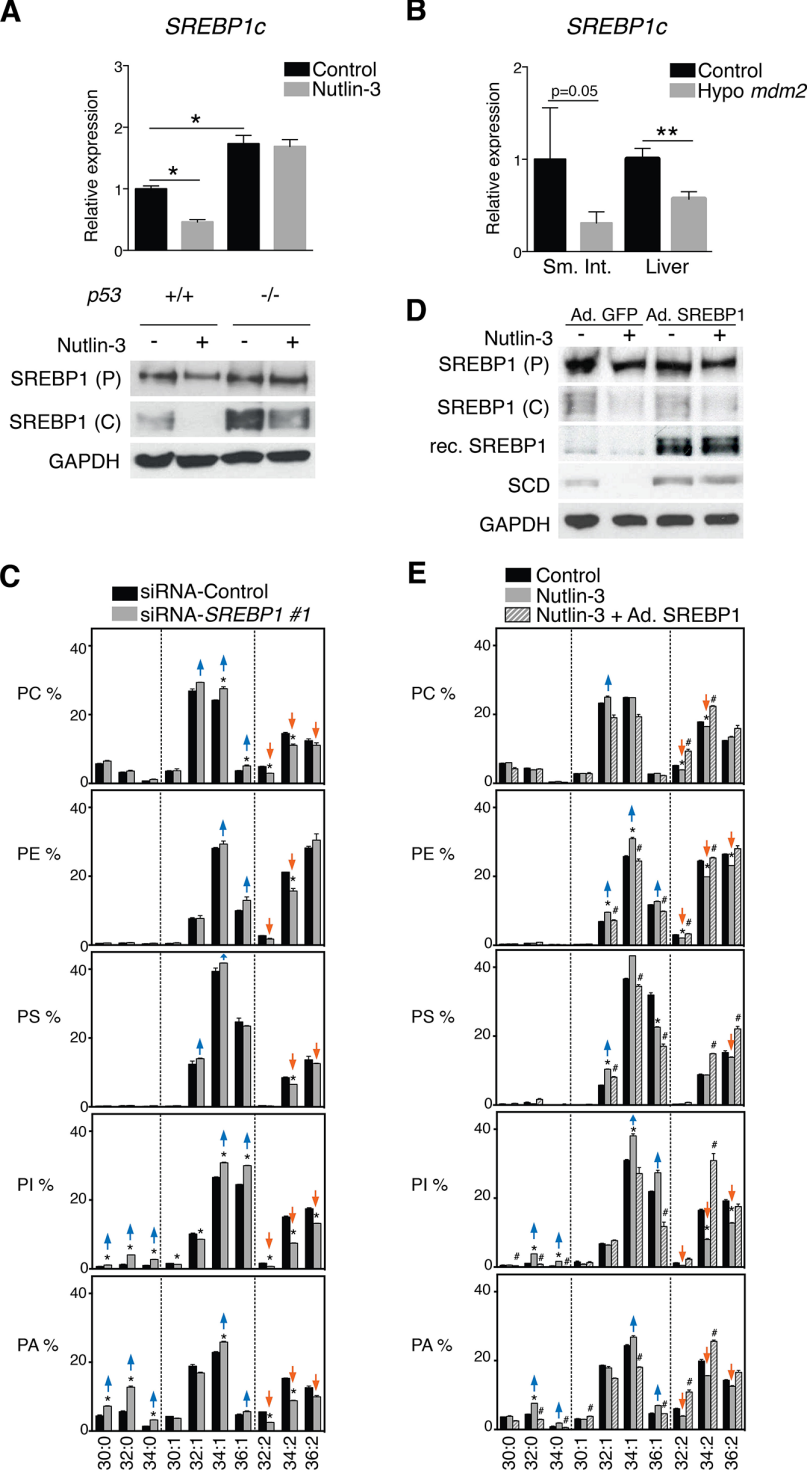
p53-induced repression of SCD is mediated by SREBP1c **A.** Activation of p53 attenuates SREBP1c expression. HCT116 *p53*^+/+^ and *p53*^−/−^ cells were treated with 5 μM nutlin-3 for 72 h. *SREBP1c* mRNA levels were assessed by RT-qPCR and expressed relative to *p53*^+/+^ cells in the control condition; *p < 0.05 by ANOVA. Levels of SREBP1 precursor (P) and cleaved (C) form were assessed by western blotting. **B.** p53 activation attenuates SREBP1c expression *in vivo*. RT-qPCR analysis of *Srebp1c* in the small intestine (Sm. Int.) and liver from hypo *Mdm2* mice relative to control mice (*n* = 3–5). **p < 0.01 by Mann-Whitney test. **C.** Effect of *SREBP1* knock-down on phospholipid profiles. HCT116 cells were transfected with siRNA *SREBP1*. After 72 h cell pellets were collected for phospholipid analysis. **D.** and **E.** Re-introduction of *SREBP1c* in Nutlin-treated cells restores SCD expression and phospholipid profiles. HCT116 *p53*^+/+^ cells were infected with an adenovirus encoding *SREBP1c* or GFP as control and were treated with nutlin-3. After 72 h, cell pellets were collected for protein or lipid analysis. P, precursor; C, cleaved, Rec, recombinant. Phospholipid graphs are organized as explained in the legend to Figure 1. *p < 0.05 (control vs nutlin-3) and #*p* < 0.05 (nutlin-3 vs nutlin-3 + Ad. SREBP1) by multiple *t* test and Holm-Sidak correction for multiple comparisons.

*SCD* is a well-known transcriptional target of the master lipogenic regulator SREBP1c, which was reported to be affected by p53 in adipocytes of obese (*ob/ob*) mice [28]. In line with these findings, nutlin-3 significantly down-regulated the expression of SREBP1c in HCT116 cells (Figure 4A). *Srebp1c* was also down-regulated in tissues of *Mdm2* hypomorphic mice (Figure 4B).

To investigate whether SREBP1c is involved in the p53-mediated changes in membrane phospholipid desaturation, we knocked-down SREBP1 using two independent siRNAs (Supplementary Figure S4). Silencing of *SREBP1c* resulted in a shift in phospholipid mono-unsaturation similar to the one observed after nutlin-3 treatment (Figure 4C and Supplementary Figure S5). Conversely, reintroduction of SREBP1c in HCT116 by infection with an adenovirus restored SCD expression in nutlin-3 treated cells (Figure 4D) and reversed the phospholipid profiles (Figure 4E). These findings show that p53 regulates fatty acid desaturation in cancer cells through downregulation of SREBP1c and its target SCD.

Consistent with the involvement of this lipogenic transcription factor, exposure of HCT116 cells to nutlin-3 evoked significant changes also in other SREBP1c targets, including ELOVL6 (ELOVL fatty acid elongase 6), *p* = 0.0055 and FADS2 (fatty acid desaturase 2), *p* = 0.0136. Also FASN (fatty acid synthase) and ELOVL5 (ELOVL fatty acid elongase 5) showed a trend towards down-regulation (Supplementary Figure S6A). In agreement with these changes, additional alterations in lipid profiles were observed, including changes in fatty acyl elongation (Supplementary Figure S6B). Overall these effects were less pronounced than the changes in mono-unsaturation. Also the total amount of phospholipids was affected by nutlin-3 treatment, suggesting additional effects on lipid metabolism (Supplementary Figure S6C).

### Repression of SREBP1c and SCD by p53 is p21-dependent

**Figure 5:**
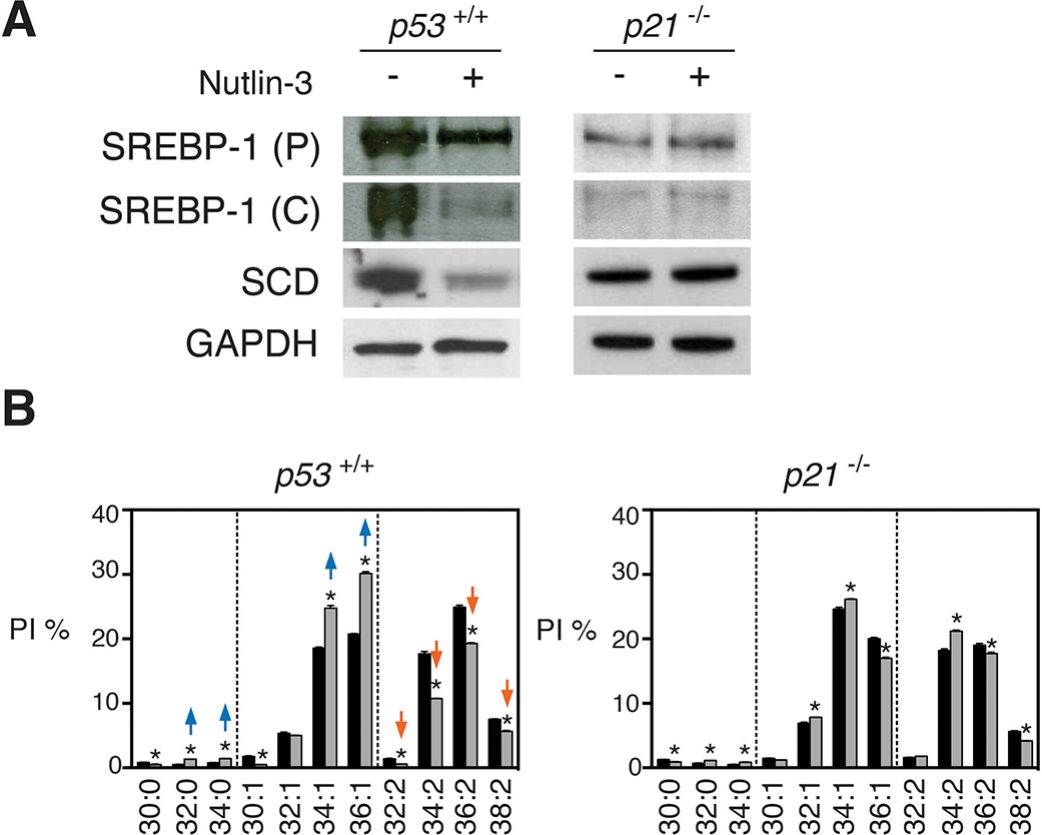
p53-induced changes in SREBP1c, SCD and membrane phospholipids are partially dependent on p21 **A.** Absence of p53-induced repression of SREBP1c and SCD in *p21*^−/−^ cells. HCT116 *p53*^+/+^ and *p21*^−/−^ cells were treated with nutlin-3 at 5 μM for 72 h and levels of SREBP1 precursor (P) and cleaved (C) form and SCD were assessed by western blotting. **B.** Effect of nutlin-3 on PI species of HCT116 *p53*^+/+^ and *p21*^−/−^ cells. *p < 0.05 (control vs nutlin-3) by multiple *t* test and Holm-Sidak correction for multiple comparisons. Data are presented as means ± SEM. Lipid profiles of HCT116 *p53*^+/+^ are the same as in Figure 1.

p53 mainly functions as a transcriptional activator [29]. Repression of genes is in most cases mediated by p21 which prevents phosphorylation of the retinoblastoma (Rb) protein maintaining E2F-regulated genes in a repressed state [6]. Consistent with this concept, HCT116 cells with a p21 knockout (*p21*^−/−^ cells) did not show a reduction of SREBP1c or SCD after nutlin-3 treatment (Figure 5A). Moreover, changes in phospholipid mono-unsaturation of HCT116 *p21*^−/−^ cells were less pronounced than in HCT116 *p53*^+/+^ after nutlin-3 treatment, as illustrated for PI (Figure 5B). These results suggest that p21 largely mediates the effects of p53 on membrane phospholipid desaturation. Consistent with earlier reports that the Rb/E2F axis downregulates SREBP1 and SREBP2 in AC61 and MEFs cells [30], E2F binding sites could be discerned in the promoter region of SREBF1 *in silico* using the ENCODE software [31] (Supplementary Figure S7). Therefore, our data support a role for the p21-Rb-E2F transcriptional network in the p53-mediated suppression of SREBP1c and SCD.

### p53-induced SCD repression attenuates AKT activation and contributes to the effects of p53 on cell survival

**Figure 6:**
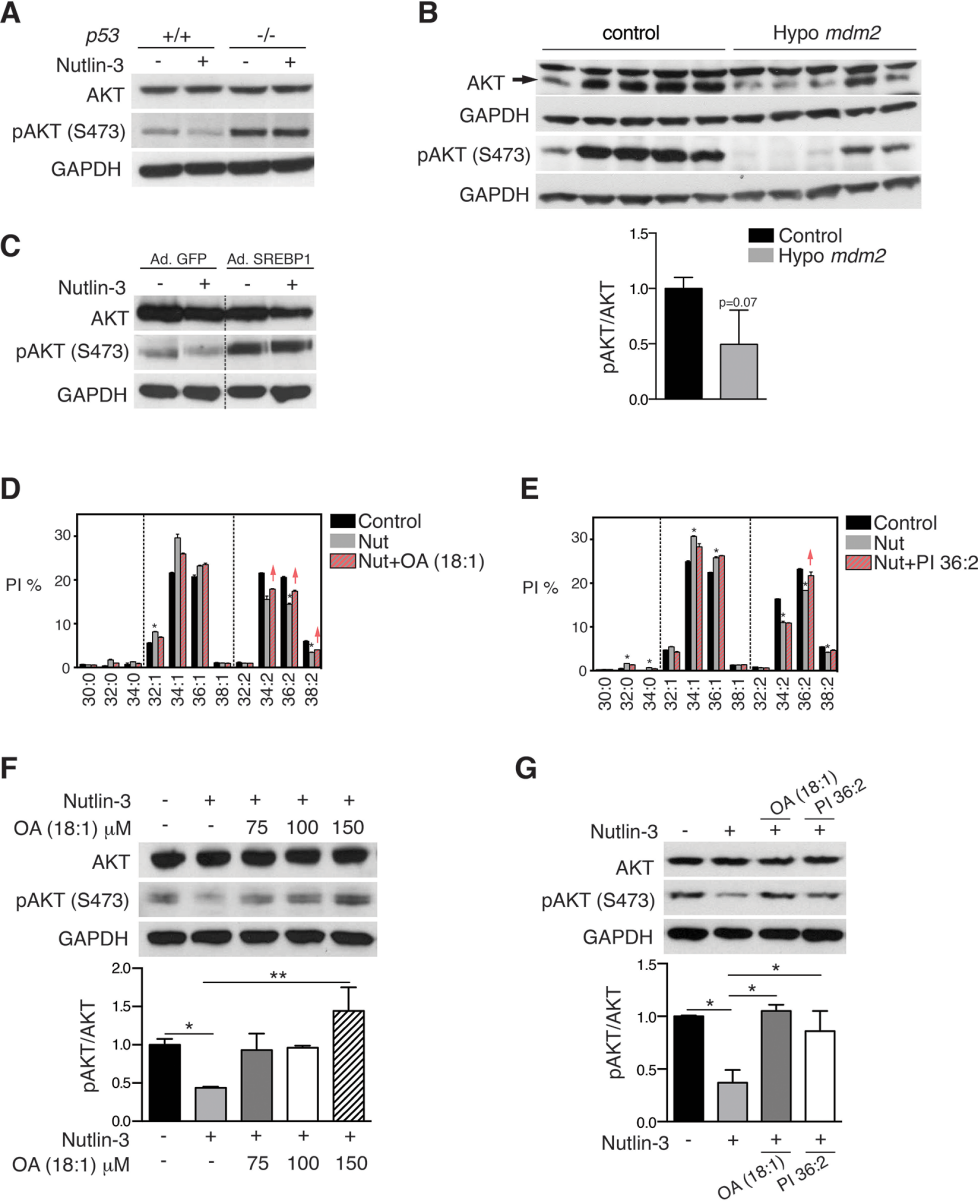
p53-mediated SCD repression attenuates AKT activation **A.** HCT116 *p53*^+/+^ and *p53*^−/−^ cells were treated with 5 μM nutlin-3 for 72 h and pAKT (S473) and total AKT levels were assessed by western blotting. GAPDH was used as loading control. **B.** AKT and pAKT (S473) levels in tissues from hypo *Mdm2* and control mice (*n* = 5). The ratio pAKT/AKT is expressed relative to control, *p* = 0, 07, unpaired *t* test. **C.** Re-introduction of SREBP1 restores pAKT in nutlin-3-treated cells. HCT116 *p53*^+/+^ cells were infected with an adenovirus encoding SREBP1c or empty virus. Four h after infection, cells were treated 5 μM nutlin-3 for 72 h. pAKT and total AKT levels were assessed by western blotting. The blot was cropped to show the conditions of interest. **D** and **F.** Oleic acid (OA) rescues the effect of p53 on pAKT by restoring phospholipid profiles. HCT116 cells were treated with OA (100 μM) in combination with 5 μM nutlin-3 (Nut). Phospholipid profiles are presented as described in the legend to Figure 1. Representative blots of total AKT and pAKT (S473) are shown. The graph presents the average pAKT/AKT ratio of two independent samples. *p < 0.05, **p < 0.01 by ANOVA. **E** and **G.** PI36:2 restores pAKT (S473) levels in nutlin-3-treated cells. HCT116 cells were treated with 5 μM nutlin-3 alone (Nut) or in combination with PI36:2 at 10 μM. After 72 h, cells pellets were collected for lipid or western blot analysis. Phospholipid profiles were presented as described in the legend to Figure 1. Panel depicts representative blots of total AKT and pAKT (S473). The graph shows the average pAKT/AKT ratio of two independent samples. *p < 0.05 by ANOVA.

According to the literature, SCD inhibition attenuates activation of the prosurvival pathway AKT [32]. Hence we determined whether SCD-induced changes in phospholipid mono-unsaturation play a role in the crosstalk between p53 and AKT. Western blot analysis confirmed that nutlin-3 treatment reduced AKT phosphorylation (pAKT) in wild-type HCT116 cells but not in *p53*^−/−^ cells (Figure 6A). Changes in pAKT over total AKT were also observed *in vivo* in tissues from *Mdm2* hypomorphic mice compared to wild type mice (Figure 6B). Consistent with the involvement of the SREBP1/SCD axis in the attenuation of pAKT by p53, infection with a SREBP1 adenovirus rescued the nutlin-3-mediated effect on pAKT (Figure 6C). Moreover, supplementation with oleic acid, the main end product of SCD, largely restored p53-induced effects on PI species (Figure 6D) and dose-dependently reversed the effect on AKT activation in cells treated with nutlin-3 (Figure 6F). In view of the prominent effect of p53 on mono-unsaturation of PI species and the strong involvement of PI species in AKT activation, we also supplemented HCT116 cell cultures with PI 36:2, one of the phospholipid species down-regulated by p53. Lipidomics analysis verified the incorporation of this PI (Figure 6E). Interestingly, restoring the levels of this single lipid species PI 36:2 largely rescued the effect of nutlin-3 on AKT activation (Figure 6G). Similar effects were observed in another independent cell line, LNCaP (Supplementary Figure S8).

**Figure 7:**
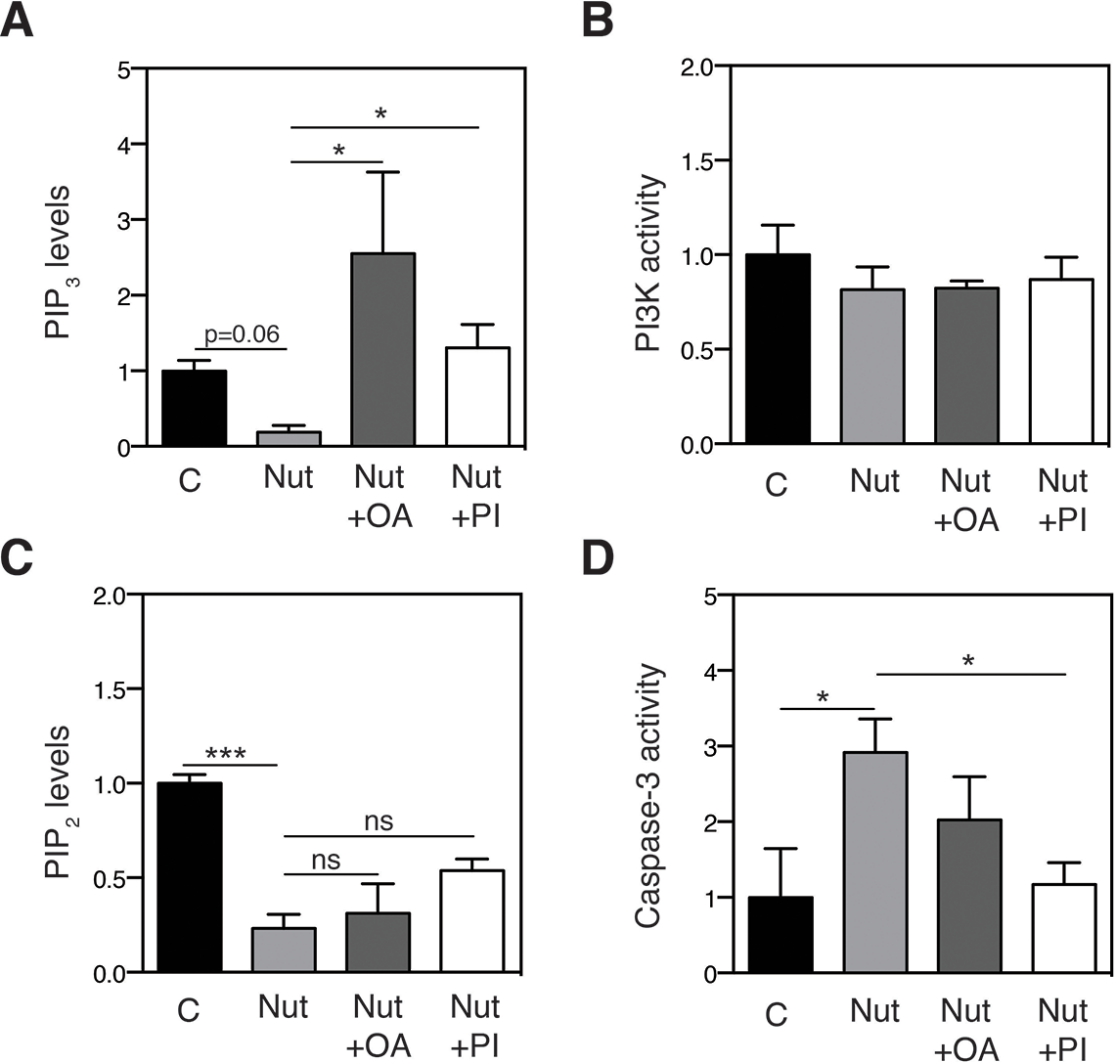
Effect of nutlin-3 and mono-unsaturated lipids on PIP_2_ / PIP_3_ levels and on cell death induction **A.** OA and PI36:2 restore PIP_3_ levels in nutlin-3-treated cells. HCT116 cells were treated with 5 μM nutlin-3 in the absence or presence of 100 μM OA or 10 μM PI36:2. After 72 h, PIP_3_ levels were measured by a PI(3, 4, 5)P_3_ Mass ELISA kit (*n* = 3). Levels are shown as fold change relative to the control group. *p < 0.05 vs nutlin-3, Kruskal-Wallis test. **B.** PI3 Kinase activity. HCT116 cells were treated with 5 μM nutlin-3 in the absence or presence of 100 μM OA or 10 μM PI36:2. After 72 h, PI3 Kinase activity was determined by measuring the PIP_3_ product with an ELISA kit (*n* = 3). **C.** PIP_2_ levels were measured using a PI(4, 5)P_2_ Mass ELISA kit (*n* = 3). Levels are shown as fold change relative to the control group. ***p < 0.001 vs nutlin-3, Kruskal-Wallis test. **D.** Cell death was measured using a caspase-3 activity kit. Caspase-3 activity is expressed as the fold change relative to the untreated control group (*n* = 3). *p < 0.05 by ANOVA. C: control, Nut: nutlin-3, Nut+OA: nutlin + Oleic acid (18:1), Nut+PI: nutlin + PI (36:2).

Since AKT activation is mediated by phosphatidylinositol-(3, 4, 5)-triphosphate (PIP_3_) [33], a phosphorylated form of PI, we next assessed the impact of p53 on PIP_3_ levels. Consistent with our hypothesis, nutlin-3 treatment attenuated PIP_3_ levels, and addition of oleic acid or PI 36:2 restored these effects (Figure 7A). The observed changes in PIP_3_ levels were not caused by altered PI3 kinase activity, as revealed by using an enzymatic assay (Figure 7B). Instead, we found that the PIP_3_ precursor, PIP_2_, was affected by nutlin-3 treatment (Figure 7C).

To demonstrate that the lipid-induced changes in AKT activation contribute to the effects of p53 on apoptotic cell death, HCT116 cells were treated with nutlin-3 in the presence or absence of oleic acid or PI 36:2 and caspase-3 activity was measured. Consistent with previous studies [35, 36], nutlin-3 treatment caused a two-fold increase in caspase-3 activity. Supplementation of cultures with oleic acid or PI 36:2 largely rescued the effect of nutlin-3 on caspase-3 mediated-apoptosis (Figure 7D). These data indicate that p53-induced changes in PI lipid species contribute to the p53-mediated control of cell survival.

## DISCUSSION

Development and progression of cancer is a complex process that typically involves the coordinated action of oncogenes and tumor suppressors. These factors form a vast network of crosstalk that remains incompletely understood. Here we provide evidence for a central role of phospholipid metabolism in the coordination of two of the most important oncogenic pathways: p53 and AKT. Using two complementary models, i.e. cancer cells treated with nutlin-3 as clinically relevant paradigm of pharmacological reactivation of p53 in cancers, and *Mdm2* hypomorphic mice as *in vivo* model of normal p53 functioning, we have demonstrated that activation of p53 evokes major changes in cellular phospholipid profiles. We have shown a striking decrease in mono-unsaturation of common phospholipid acyl chains, particularly of PI. Using knockdown, chemical inhibition as well as forced re-introduction, we could provide evidence that these changes in phospholipid profiles are largely caused by p53-induced down-regulation of SCD, the main enzyme involved in mono-unsaturation. Consistent with previous findings that SCD is a target of SREBP1 [37, 38] and, in line with the observation that p53 down-regulates SREBP1 in adipocytes from obese (*ob/ob*) mice [28], we provide evidence that down-regulation of SCD occurs downstream of p53-induced SREBP1 repression in our models. Since p53 acts mainly as a transcriptional activator, and gene repression is usually caused by indirect effects mediated by the p21/Rb/E2F pathway [29, 39], it is reasonable to speculate that p53-dependent SREBP repression may involve this pathway. The lack of p53-binding sites in the SREBP1 promoter region in existing ChIP-seq data [40, 41], the presence of E2F binding sites, and the lack of p53-mediated changes in mono-unsaturation in p21 knockout cells give support to this concept. To exclude that the effects on membrane phospholipids were not a mere consequence of p21-dependent cell cycle arrest and/or apoptosis, membrane lipid analysis was carried out in confluent cell cultures and floating death cells were removed before lipid analysis. Other additional mechanisms linking p53 to lipid metabolism are however feasible and are emerging. Together, these findings implicate a novel cascade mechanism whereby p53, via the p21/Rb/E2F pathway, represses SREBP1 expression, resulting in down-regulation of SCD and a subsequent decrease in mono-unsaturation of phospholipid acyl chains. Conversely to the wild-type p53-dependent repression of SCD, loss of p53 resulted in an increased expression of SCD in HCT116 *p53*^−/−^ cells. Not surprisingly, many tumors, most of which have an impaired p53 pathway, have shown overexpression of SCD [42, 43] and elevated levels of mono-unsaturated phospholipid species [44, 45]. With respect to the downstream involvement of phospholipid mono-unsaturation in p53-mediated biological effects on cancer cell biology, we have uncovered a novel regulatory mechanism by which p53, through modulation of mono-unsaturation of phospholipid acyl chains, affects the activation of AKT, one of the most commonly activated oncogenes [46]. This conclusion is in line with previous reports that SCD affects the phosphorylation of AKT [32] and is supported by our observation that addition of oleic acid reverses the p53-induced repression of AKT phosphorylation. In support of the role of PI metabolism in this effect, altered mono-unsaturation of lipids was most pronounced in the PI headgroup class. Furthermore, modulation of p53 caused major changes in the levels of PIP_3_, the main regulator of AKT activation, and highly specific supplementation with only one phospholipid species, PI 36:2, one of the main phospholipids affected by p53, largely rescued PIP_3_ levels and pAKT activation. With respect to how mono-unsaturation affects PIP_3_ levels and AKT activation, we provided evidence that PI3 kinase activity is not affected but that rather PIP_2_, the main precursor for PIP_3_, is affected by p53. This is in line with previous reports that PIs with mono-unsaturated acyl chains are better substrates for enzymes involved in PIP_2_ synthesis than saturated ones [34]. Consistent with a link with the AKT pathway, we have shown evidence that the p53-induced effect on lipid mono-unsaturation is involved in p53-mediated modulation of cell survival. In view of previous reports, it may be interesting to explore to what extent this mechanism is also implicated in other p53-mediated functions including cellular senescence [47], and/or the choice between senescence and quiescence upon p53 activation, which depends on its ability to inhibit the mTOR pathway [48].

**Figure 8:**
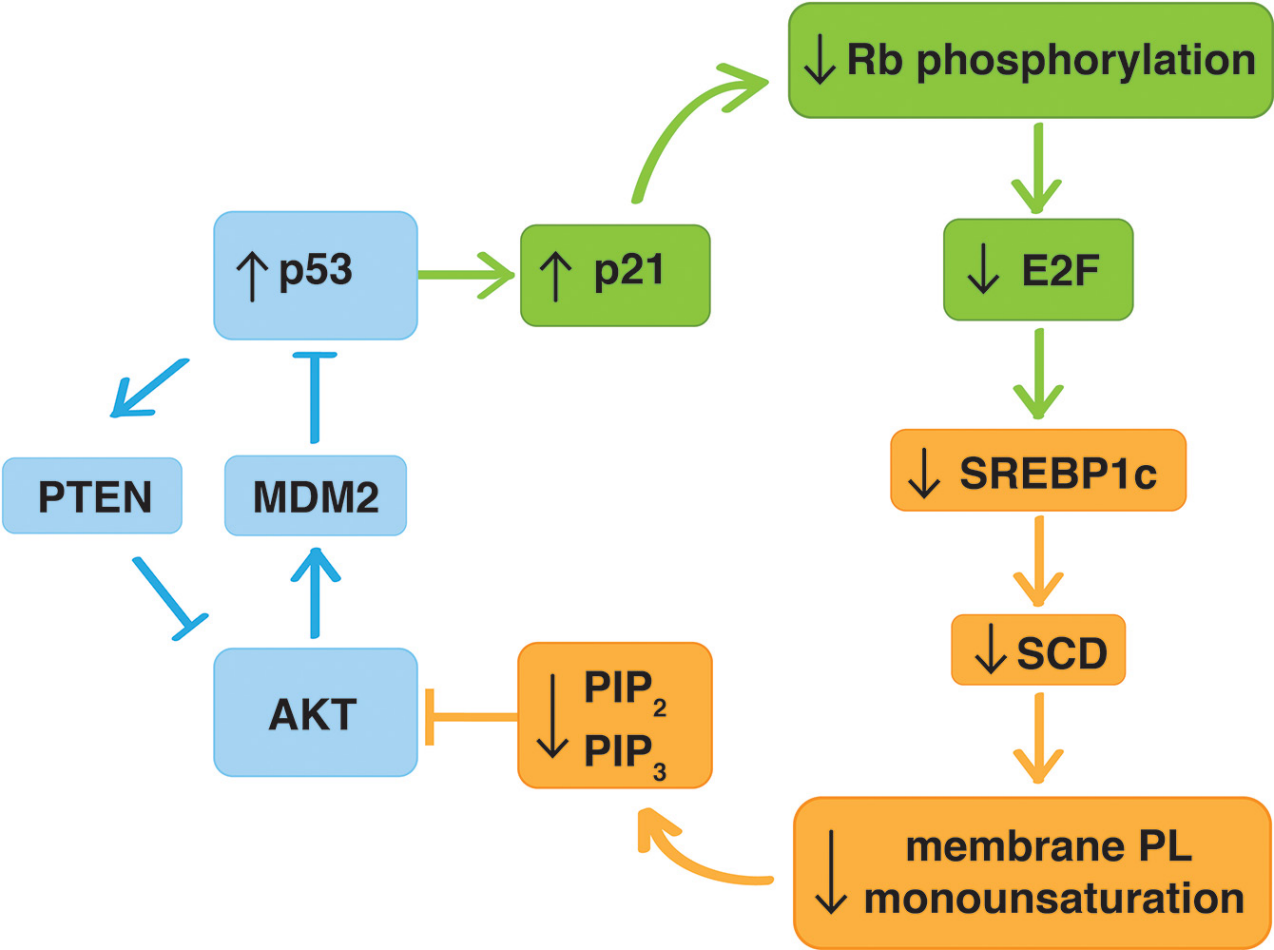
Graphical overview of the mechanism underlying p53-regulation of the AKT pathway through modulation of phospholipid mono-unsaturation In addition to the previously reported p53-AKT crosstalk involving AKT-induced activation of MDM2 and p53-mediated induction of the phosphatase and tensin homolog (PTEN) (blue), p53 affects AKT activation through a novel indirect mechanism involving activation of the p53-target p21, which prevents phosphorylation of the retinoblastoma (Rb) protein maintaining E2F-regulated genes in a repressed state (green). This leads to down-regulation of the expression of SREBP1c and its downstream target SCD, resulting in a decrease in mono-unsaturation of membrane phospholipids, decreased PIP_2_ and PIP_3_ levels, and ultimately decreased phosphorylation and activation of AKT (orange). PTEN (phosphatase and tensin homolog), MDM2 (murine double minute 2), AKT (v-akt murine thymoma viral oncogene), p21 (cyclin-dependent kinase inhibitor 1A), Rb (retinoblastoma), E2F (E2F transcription factor), SREBP1c (sterol regulatory element bindingprotein-1c), SCD (stearoyl CoA desaturase 1), PL (phospholipid), PIP_2_ (phosphatidylinositol-(4, 5)-triphosphate), PIP_3_ (phosphatidylinositol-(3, 4, 5)-triphosphate).

Collectively our data provide an alternative mechanism of p53-AKT crosstalk. In addition to the previously reported links involving AKT-mediated activation of MDM2 and the p53-mediated induction of the phosphatase and tensin homolog (PTEN) [49, 50], we propose that p53, through an indirect mechanism involving the p21/Rb/E2F pathway, represses the expression of SREBP1c and its downstream target SCD. The resulting change in mono-unsaturation of membrane phospholipids alters PIP_2_ and PIP_3_ levels, and ultimately affects the phosphorylation and activation of AKT (Figure 8). To what extent this novel mechanism contributes to this crosstalk *in vivo*, relative to the other mechanisms, remains to be explored in appropriate transgenic models. If confirmed, this novel mechanism provides novel opportunities to enhance the efficacy of strategies targeting these key oncogenic pathways by pharmacological intervention of lipid metabolism. Recent reports on the modulation of SREBP1 and/or SCD in tumor models support the potential of this strategy [23, 24, 43, 51].

## MATERIALS AND METHODS

### Animals

Transgenic *Mdm2* hypomorphic C57BL/6J mice expressing elevated levels of p53 [26] were obtained from Dr. M E Perry from the National Cancer Institute, National Institutes of Health (Bethesda, Maryland, USA). Wild type C57BL/6J mice, purchased from the R. Janvier Breeding center (Le Genest St. Isle, France), were used as control. Male mice 6–7 week-old were used for all studies. Organs were collected snap frozen for RNA, proteins and lipid analysis. All animal experiments were performed in accordance to institutional guidelines and approved by the Ethical Committee of the KU Leuven.

### Cell culture and reagents

HCT116 *p53*^+/+^, HCT116 *p53*^−/−^ and HCT116 *p21*^−/−^ were kindly provided by Dr. B Vogelstein from Johns Hopkins University (Baltimore, MD, USA). Cells were maintained in Dulbecco's modified Eagle's medium containing 10% fetal bovine serum (FBS), penicillin-streptomycin, sodium pyruvate and non-essential amino acids. LNCaP were obtained from the American Type Culture Collection (Manassas, Virginia, USA) and cultured in RPMI 1640 medium supplemented with 10% FBS. Cells were treated with 5 μM nutlin-3 from a stock of 10 mM in EtOH 100% or with 0.5 μM doxorubicin obtained from Sigma (St Louis, Missouri, USA). Cells were treated at high cell density to minimize putative confounding effects of the treatment on the cell cycle. The medium was refreshed every day with addition of new compound. The SCD inhibitor, 4-(2-Chlorophenoxy)-N-(3-(3-methylcarbamoyl)phenyl)piperidine-1-carboxamide, was obtained from BioVision (Milpitas, California, USA) and stored as a 2, 58 mM stock solution in DMSO at −20°C. Oleic acid (Sigma) was dissolved in EtOH 100% as a 100 mM stock solution and bound to fatty acid-free bovine serum albumin (BSA) (Sigma) in a 4% BSA solution in 0, 9% NaCl. Fatty acid/BSA complexes were incubated at 37°C for 1 h to obtain a final concentration of 1, 25 mM and added to the culture medium at the indicated concentrations. Phosphatidylinositol (PI) 36:2 was purchased from Avanti (Alabaster, Alabama, USA) Polar Lipids (850149) and stored as a 10 mM stock solution in EtOH100%.

### Adenoviral infection

Cells were infected with an adenovirus encoding human SCD obtained from Vector Biolabs (Malvern, Pennsylvania, USA), catalog number ADV- 222321 or the first 403 amino acids of SREBP1c (kindly provided by Dr. Fabienne Foufelle, Université Paris, Paris, France). A control virus (empty or encoding GFP) was used as negative control.

### RNA interference

Small interfering RNAs (siRNAs) targeting *SCD* (LQ-005061-00-0005 ON-Target plus set of 4), *SREBF1* (LQ-006891-00-0005 ON-Target plus set of 4) and control siRNA (D-001810-01-20 ON-Target plus Non-targeting siRNA#1) were obtained from Dharmacon (Lafayette, Colorado, USA) and reverse transfected at 5 nM using lipofectamine siRNA reagent purchased from Invitrogen (Carlsbad, California, USA). Forty-eight hours after transfection, cells were collected for RNA and protein analysis to check gene knockdown. Two independent siRNAs for each gene were selected for further lipid analysis.

### Quantitative RT-qPCR

Total RNA was extracted from cells and mouse tissues using the PureLink TM Total RNA purification system from Life Technologies (Carlsbad, California, USA). One μg was converted to cDNA using Superscript II RT and random hexamer primers (Invitrogen). Quantitative real-time PCR was performed on a 7500 Fast Real-Time system from Applied Biosystems (Carlsbad, California, USA). Expression levels were normalized against RPL13A or 18S. Oligonucleotide primer sequences were taken from PrimerBank (mouse) or designed with Primer3plus (human) and synthesized by IDT (Integrated DNA Technologies, Coralville, Iowa, USA). The following primer sets were used: human *p21* (Fwd, 5′ AGCAGAGGAAGACCATGTGGA-3′, Rev, 5′-AATCTGTCATGCTGGTCTGCC-3′), human *SCD* (Fwd, 5′-CCGGGAGAATATCCTGGTTT-3′, Rev, 5′-GCGGTACTCACTGGCAGAGT-3′), human *SREBP1c* (Fwd, 5′-TGCTGACCGACATCGAAGGTGAA-3′, Rev, 5′-TTCGAAAGTGCAATCCATGGCTCC-3′), human *ELOVL6* (Fwd, 5′-CAAAGCACCCGAACTAGGAG-3′, Rev, 5′-TGGTGATACCAGTGCAGGAA-3′), human *FADS2* (Fw, 5′-ATCCCTTTCTACGGCATCCT-3′, Rev, 5′-TGGCTACTGAACCAGTCACG-3′), human FASN (Fwd, 5′-TCCGAGATTCCATCCTACGC-3′, Rev, 5′-GCA GCTGTGACACCTTCAGG-3′), human *ELOVL5* (Fwd, 5′-GTGCACATTCCCTCTTGGTT-3′, Rev, 5′-TTCA GGTGGTCTTTCCTTCG-3′), human *RPL13A* (Fwd, 5′-C CTGGAGGAGAAGAGGAAAGAGA-3′, Rev, 5′-TTGAG GACCTCTGTGTATTTGTCAA-3′), mouse *p21* (Fwd, 5′- CCTGGTGATGTCCGACCTG-3′, Rev 5′-CCATGAGCG CATCGCAATC-3′), mouse *Scd* (Fwd, 5′-TTCTTGCGATA CACTCTGGTGC-3′, Rev 5′-CGGGATTGAATGTTCTT GTCGT-3′), mouse *Srebp1c* (Fwd, 5′-GCAGCCACCATC TAGCCTG-3′, Rev 5′- CAGCAGTGAGTCTGCCTTG AT-3′) and *18S* (Fwd, 5′-CGCCGCTAGAGGTGAAA TTC-3′, Rev, 5′-TTGGCAAATGCTTTCGCTC-3′).

### Western blotting

Cells were lysed in a reducing NuPage sample loading buffer (Invitrogen). Equal amounts of proteins were separated onto precast gels (NuPAGE, Invitrogen) and transferred to Hybond TM nitrocellulose membrane obtained from GE Healthcare (Pittsburgh, Pennsylvania, USA). Blots were blocked in Tris buffered saline with 0, 1% Tween-20 (TBST) and 5% nonfat dry milk and incubated overnight with the appropriate primary antibody. The following primary antibodies and dilutions were used: human p53 from Santa Cruz Biotechnology (Dallas, Texas, USA), sc-6243 1:500; human p21 (Santa Cruz Technology, sc-6246 1:500); human MDM2 (Santa Cruz Biotechnology, sc-965 1:500); human SCD from Cell Signaling (Beverly, Massachusetts, USA), M38 1:1000); human SREBP1 from Active Motif (Carlsbad, California, USA), 39939 1:1000); anti AKT/PKB (Invitrogen, 44–609G 1:1000), Phospho-AKT (Ser473), (Invitrogen, 44–621G 1:1000) and GAPDH (Invitrogen 1:10000). Immunoreactive proteins were detected with the Enhanced Chemiluminescence Western Blotting Detection System from Amersham Biosciences (Pittsburgh, Pennsylvania, USA).

### Mass spectrometry-based lipidomics

Cells were washed in cold PBS and collected by scraping. Mouse tissues were disintegrated in a tissue grinder homogenizer in cold PBS. Lipids were extracted using a modified Bligh-Dyer protocol and phospholipids were analyzed by electrospray ionization tandem mass spectrometry (ESI-MS/MS) on a hybrid triple quadrupole linear ion trap mass spectrometer (4000 QTRAP, AB SCIEX, Framingham, Massachusetts, USA) operated in MRM mode and equipped with a robotic sample injection and ionization device (TriVersa NanoMate, Advion) as described [19]. To quantify the total amount of phospholipids, we summed the abundances of individually measured species within each phospholipid class and normalized based on the amount of DNA. Acyl chain composition of individual PI species was determined by reversed phase liquid chromatography-MS/MS using a QTOF mass spectrometer (Agilent 6530, Palo Alto, USA) as described in [52].

### Caspase-3 activity

HCT116 cells were lysed in a buffer containing 100 mM Hepes pH 7.4, 10% sucrose, 1% Triton X100, 2.5 mM EDTA, 5 mM DTT, 1 mM PMSF, 2 μg/ml pepstatin and 2 μg/ml leupeptin, and incubated on ice for 30 min. Cell debris was pelleted and caspase activity was determined on the supernatant fractions by incubating 50 μg of protein with 50 μM fluorogenic caspase-3 substrate Ac-DEVD-AMC (Bachem) for 45 min at 37°C. Levels of released AMC were quantified on a FlexStation spectrophotometer (excitation 360 nm, emission 460nm) operated with SoftMax Pro software (Molecular Devices, California, USA).

### PI(3,4,5)P_3_ and PI(4,5)P_2_ measurements

Cells were seeded in 75 cm^2^ or 175 cm^2^ flasks and treated as indicated for 72 h. Neutral lipids were extracted and PIP_2_ and PIP_3_ levels measured using PIP_2_ and PIP_3_ mass ELISA kits from Echelon (Salt Lake City, Utha, USA), catalog numbers K-4500s and K-2500s, respectively.

### PI3 kinase assay

5 × 10^5^ cells were seeded in 10 cm dishes and treated as indicated for 72 h. PI3K was isolated by immunoprecipitation using an antibody to the PI3K-p85 subunit from Cell Signaling (Beverly, Massachusetts, USA), catalog number 4292. PI3K reactions were allowed to proceed for 3 hours and the ability of PI3K to convert exogenous PIP_2_ to PIP_3_ was evaluated by measuring the generated PIP_3_ using a competitive ELISA kit from Echelon (Salt Lake City, Utha, USA), catalog number K-1000s.

### ChIP-seq analysis in ENCODE

To test whether SREBF1 could be a E2F target gene, we have assessed the presence of E2F ChIP peaks around the SREBF1 TSS from multiple cell types and for different E2F family members using data available in the ENCODE project [31].

### Statistical analysis

Data are expressed as means ± SEM. Differences between two groups were assessed using the *t* test or Mann-Whitney test depending on the parametric or non parametric distribution of the sample, respectively. For determining differences between more than two groups, the ANOVA with Holm-Sidak post-test or the Kruskal-Wallis with Dunn's post-test were used depending on the parametric or non parametric distribution of the sample, respectively. The lipidomics data was analyzed with multiple *t* test and Holm-Sidak post-test for multiple comparisons. Differences were considered statistically significant with *p* values of < 0.05. GraphPad Prism 5.0 software (Graphpad Software, Inc.) was used for all statistical analysis.

## SUPPLEMENTARY FIGURES


