# Supplementary material for: p53 attenuates AKT signaling by modulating membrane phospholipid composition
Source: Oncotarget. 2015 Jun 3;6(25):21240–54. doi: 10.18632/oncotarget.4067 (PMC4673262; doi:10.18632/oncotarget.4067)
Supplement: Supplementary file 1 [file oncotarget-06-21240-s001.pdf]

SUPPLEMENTARY FIGURES

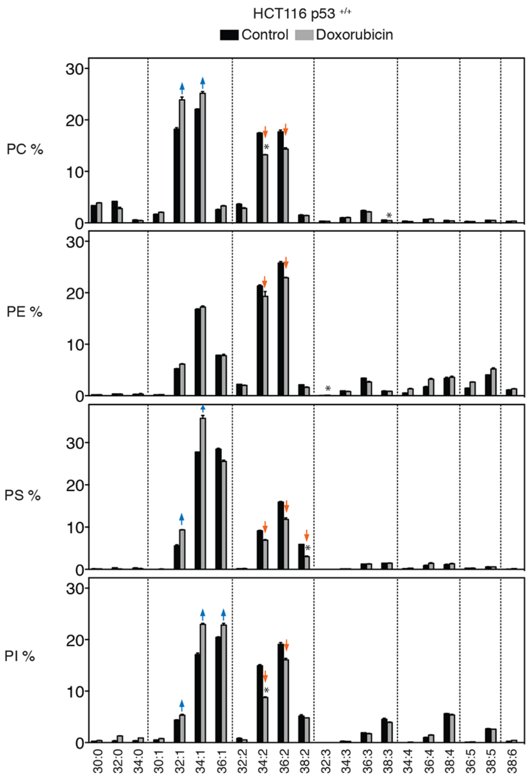

Supplementary Figure S1: Effect on the abundance of intact phospholipid species of HCT116 *p53*$^{+/+}$ cells treated with 0.5 µM of doxorubicin for 48 hours. The graph shows the relative abundance of the different measured lipid species in doxorubicin-treated and control cells. Lipid species are ordered based on the number of unsaturations in both fatty acyl chains combined, and within each unsaturation subgroup, based on the total carbon number. *$p < 0.05$ by multiple *t* test and Holm-Sidak correction for multiple comparisons. Data are presented as means ± SEM. (Phosphatidylcholine (PC), Phosphatidylethanolamine (PE), Phosphatidylserine (PS) and Phosphatidylinositol (PI)).

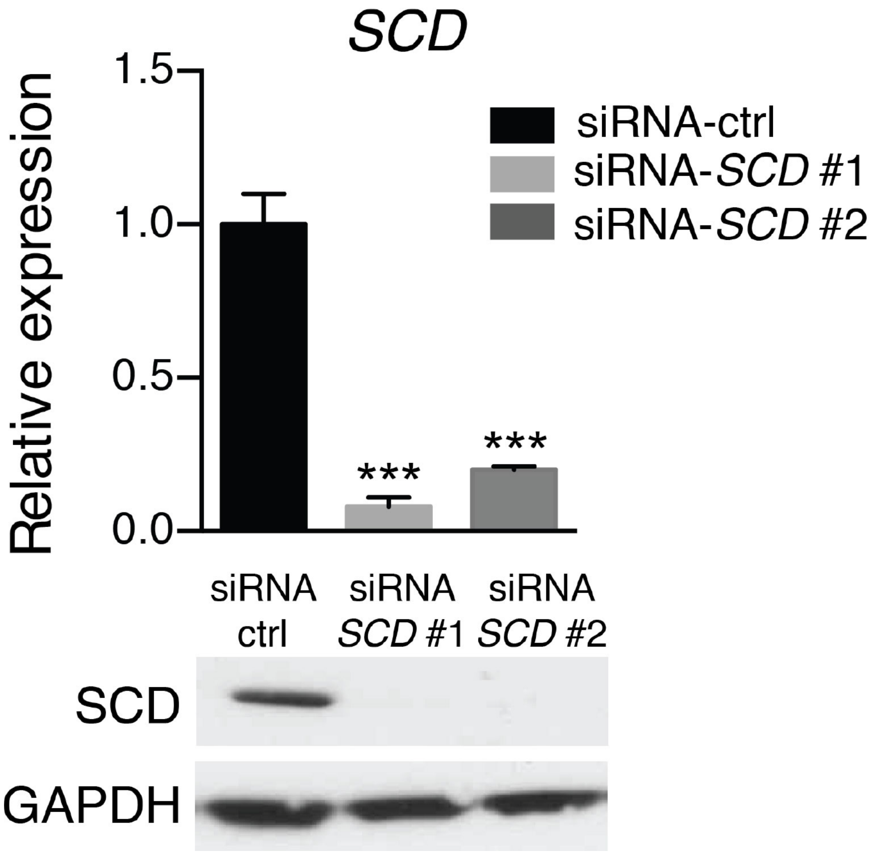

Supplementary Figure S2: *SCD* knock-down. HCT116 *p53*$^{+/+}$ were transfected with a control siRNA or siRNA targeting SCD (siRNA-*SCD* #1 and #2). Forty-eight h after transfection cells were collected for RT-qPCR and western blot analysis. ***$p < 0.001$ by Student's t test.

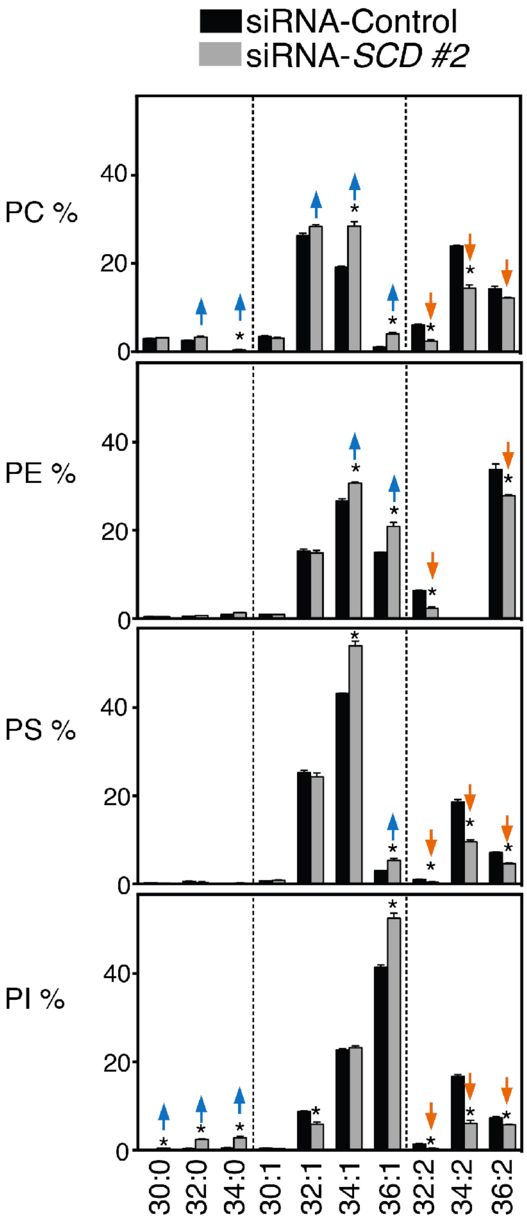

Supplementary Figure S3: Effect of *SCD* KD on lipid profiles. HCT116 cells were transfected with 5 nM of siRNA targeting *SCD (siRNA-SCD #2)***.** After 72 h, cell pellets were collected for lipid analysis. The graph shows the relative abundance of the different measured lipid species in siRNA control and siRNA SCD transfected cells. Lipid species are ordered based on the number of unsaturations in both fatty acyl chains combined, and within each unsaturation subgroup, based on the total carbon number. *$p < 0.05$ by multiple t test and Holm-Sidak correction for multiple comparisons. Data are presented as means ± SEM.

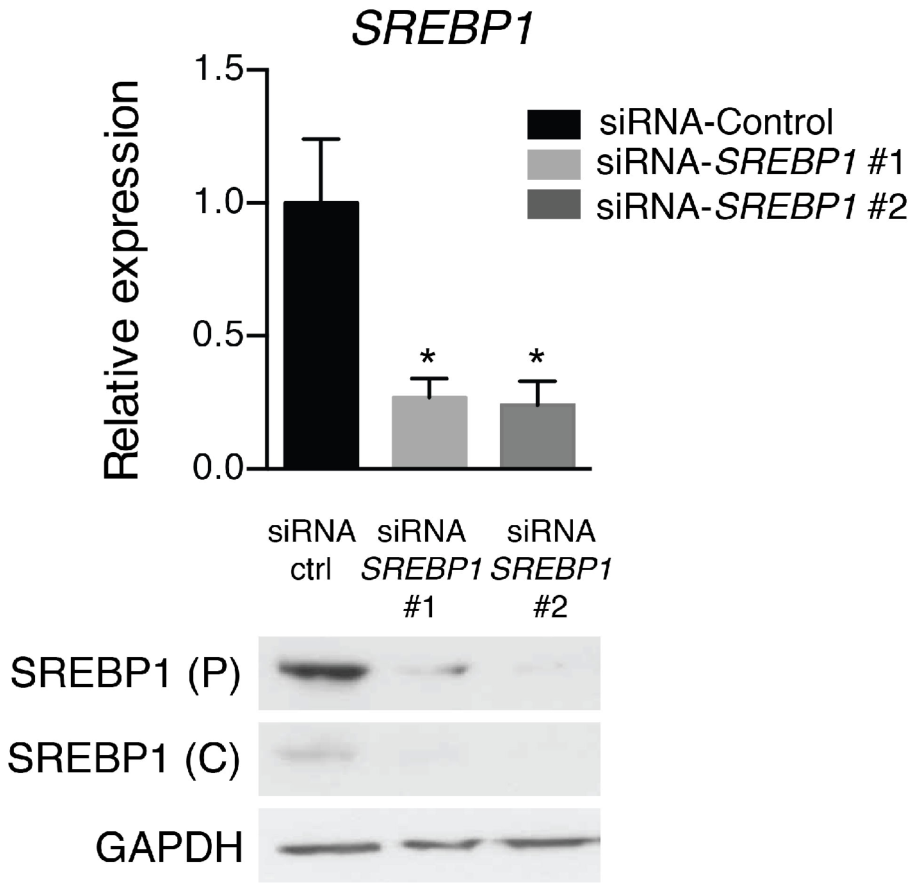

Supplementary Figure S4: *SREBP1* knock-down. HCT116 *p53*$^{+/+}$ were transfected with a control siRNA or siRNA targeting *SREBP1* (siRNA-SREBP1 #1 and #2). Forty-eight h after transfection cells were collected for RT-qPCR and western blot analysis. *$p <$ 0.05 by Student's t test.

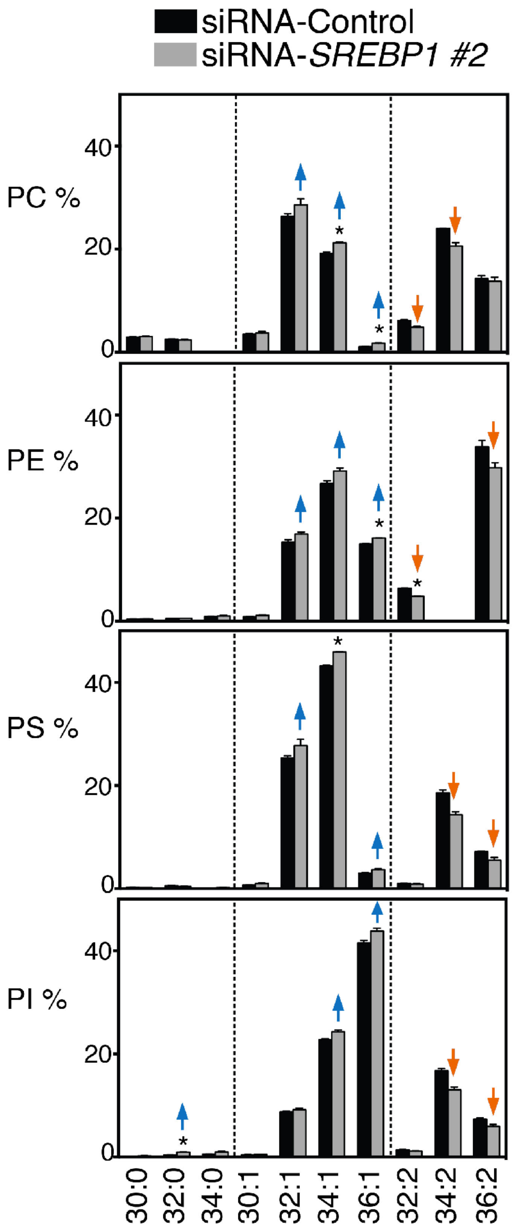

Supplementary Figure S5: Effect of *SREBP-1* KD on lipid profiles. HCT116 cells were transfected with 5 nM of siRNA targeting SREBP1 (siRNA-SREBP1 #2). After 72 h, cell pellets were collected for lipid analysis. The graph shows the relative abundance of the different measured lipid species in siRNA control and siRNA SCD transfected cells. Lipid species are ordered based on the number of unsaturations in both fatty acyl chains combined, and within each unsaturation subgroup, based on the total carbon number. *$p < 0.05$ by multiple t test and Holm-Sidak correction for multiple comparisons. Data are presented as means ± SEM.

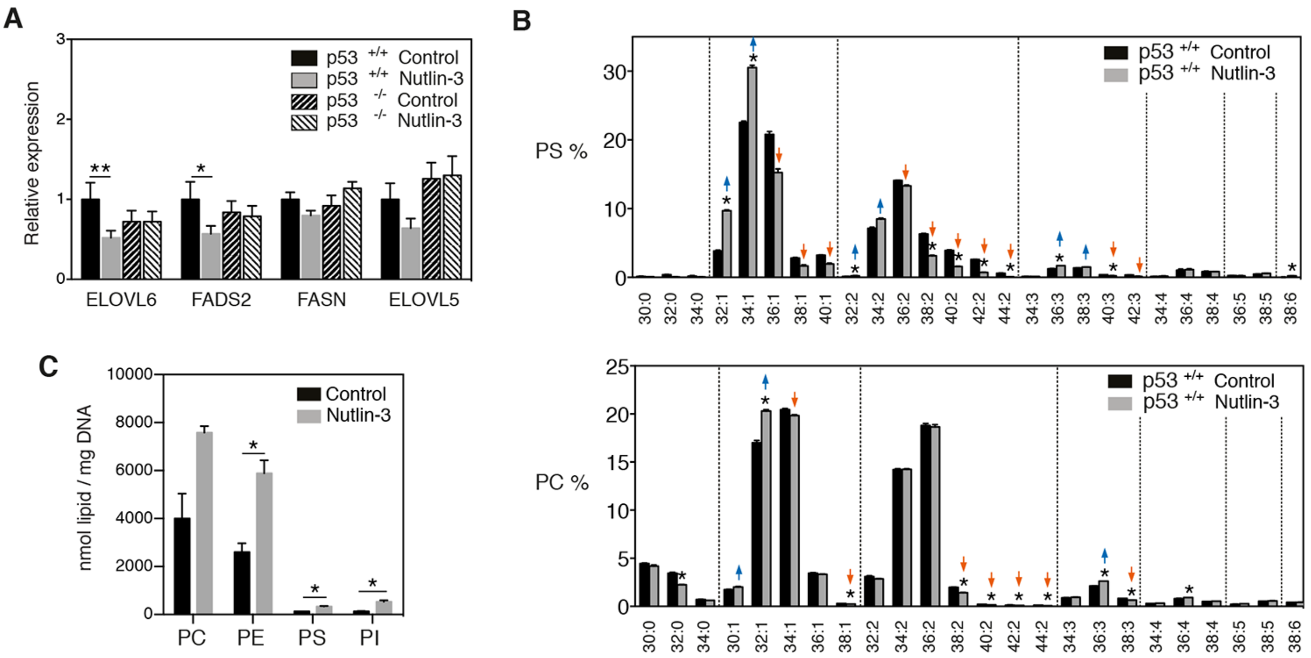

Supplementary Figure S6: A. Effect of nutlin-3 on the expression of lipogenic genes. HCT116 p53+/+ and p53–/– cells were treated with 5 µM nutlin-3 for 72 h. mRNA levels of ELOVL6 (ELOVL6 fatty acid elongase 6), FADS2 (fatty acid desaturase 2), FASN (fatty acyl synthase) and ELOVL5 (ELOVL5 fatty acid elongase 5) were assessed by RT-qPCR and expressed relative to p53+/+ cells in the control condition; *$p < 0.05$, **$p < 0.01$ by *t* test. **B.** Effect of nutlin-3 on the abundance of intact phospholipid species (Phosphatidylserine (PS) and Phosphatidylcholine (PC)) of HCT116 p53+/+ cells. The graph shows the relative abundance of the different measured lipid species in nutlin-3-treated and control cells. Lipid species are ordered based on the number of unsaturations in both fatty acyl chains combined, and within each unsaturation subgroup, based on the total carbon number. *$p < 0.05$ by multiple *t* test and Holm-Sidak correction for multiple comparisons. Data are presented as means ± SEM. **C.** Effect of nutlin-3 on the total quantity of phospholipid classes in HCT116 cells. Graph displays the abundance (nmol lipid / mg DNA) of different lipid classes: Phosphatidylcholine (PC), Phosphatidylethanolamine (PE), Phosphatidylserine (PS) and Phosphatidylinositol (PI). *$p < 0.05$ by *t* test. Data are presented as means ± SEM.

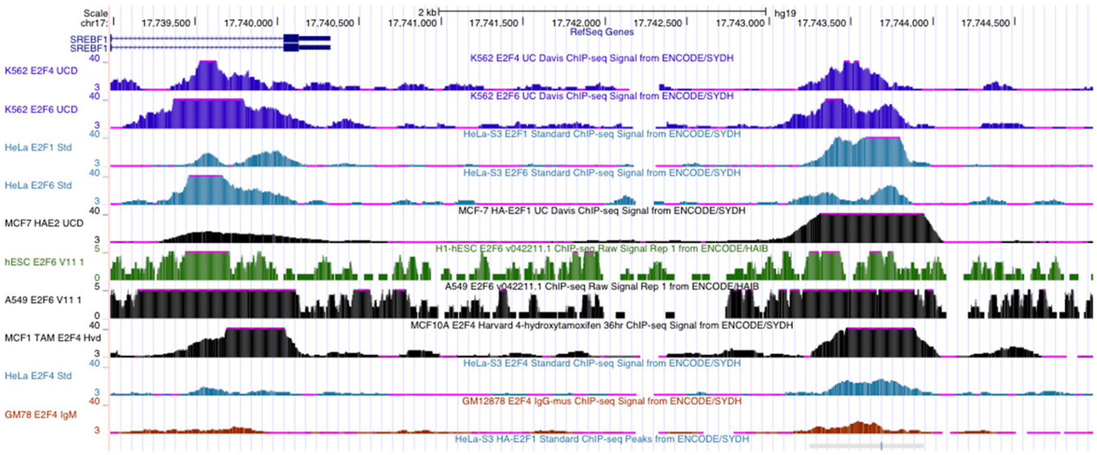

Supplementary Figure S7: ChIP-seq experiments in ENCODE. Chip-seq data generated by the ENCODE project shows E2F binding sites ± 700bp downstream and ± 3kb upstream around the SREBF1 TSS in cell lines K562, Hela, MCF7, hESC, A549, MCF10A and GM78.

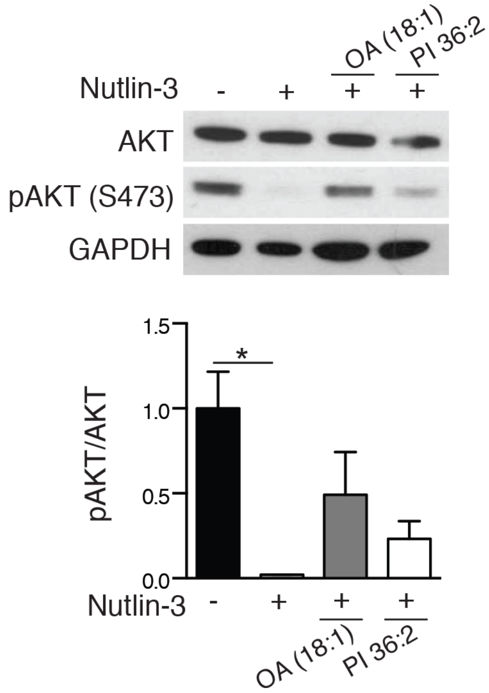

Supplementary Figure S8: Oleic acid and PI 36:2 rescue pAKT levels in nutlin-3-treated LNCaP cells. LNCaP cells were treated with 5 μM nutlin-3 alone or in combination with oleic acid 100 μM or PI 36:2 at 10 μM. After 72 h, cells pellets were collected for western blot analysis. Panel depicts representative western blot of total AKT and pAKT (S473). The graph shows the average pAKT/AKT ratio of two independent samples. *$p < 0.05$ vs nutlin-3, ANOVA.
